# Arresting Hemorrhage after Extraction of Teeth

**Published:** 1872-08

**Authors:** 


*Arresting Hemorrhage after Extraction of Teeth.*—For this purpose Dr. M. F. Shields, in the *Dental Cosmos*, recommends "Filling up the socket with a pellet of cotton, saturated with sandarac varnish. In the case of a young lady, weak and faint from the loss of blood six hours after the extraction, he applied a pellet of cotton saturated with perchloride of iron pushed into the socket, and then covered this with a large pellet saturated with the varnish, filling up the space between the teeth and the bottom of the socket. The value of this agent is due to the rapidity with which it solidifies. This property makes it easy of application, relieving the dentist from the necessity of taking impressions and fitting appliances, shortens the operation, and saves time and blood."

[A better method we conceive, and one we have been following for years past, is to saturate a pellet of cotton with sandarach varnish, and on the end of this take up a sufficient quantity of the powdered subsulphate of iron, which is carried direct to the mouths of the bleeding vessels in the alveolar cavity, immediately after this cavity has been cleansed of blood.—Ed.]

